# Multidimensional correlation of nuclear relaxation rates and diffusion tensors for model-free investigations of heterogeneous anisotropic porous materials

**DOI:** 10.1038/s41598-018-19826-9

**Published:** 2018-02-06

**Authors:** João P. de Almeida Martins, Daniel Topgaard

**Affiliations:** Division of Physical Chemistry, Department of Chemistry, Lund University, Lund, Sweden

## Abstract

Despite their widespread use in non-invasive studies of porous materials, conventional MRI methods yield ambiguous results for microscopically heterogeneous materials such as brain tissue. While the forward link between microstructure and MRI observables is well understood, the inverse problem of separating the signal contributions from different microscopic pores is notoriously difficult. Here, we introduce an experimental protocol where heterogeneity is resolved by establishing 6D correlations between the individual values of isotropic diffusivity, diffusion anisotropy, orientation of the diffusion tensor, and relaxation rates of distinct populations. Such procedure renders the acquired signal highly specific to the sample’s microstructure, and allows characterization of the underlying pore space without prior assumptions on the number and nature of distinct microscopic environments. The experimental feasibility of the suggested method is demonstrated on a sample designed to mimic the properties of nerve tissue. If matched to the constraints of whole body scanners, this protocol could allow for the unconstrained determination of the different types of tissue that compose the living human brain.

## Introduction

Magnetic Resonance Imaging (MRI) has been firmly established as the method of choice for non-invasive investigations of the structure of the living human brain. In its simplest form, MRI yields maps of the ^1^H spin density weighted by nuclear relaxation. The detected signal originates mainly from water because of its high concentration and favourable relaxation properties. The longitudinal relaxation rate *R*_1_ of water is exquisitely sensitive to the local chemical composition of the tissue^1–6^ while the transverse relaxation rate *R*_2_ additionally carries information about micro- and mesoscale tissue structures^7–9^. The sensitivity of *R*_1_ and *R*_2_ to a plethora of tissue properties is a great advantage when aiming for contrast between tissue types and detection of subtle pathological changes, but it is also a challenge when attempting to interpret the observed contrast in terms of a specific property. Diffusion MRI, on the other hand, has a more direct relation between tissue microstructure and the measurable parameters, such as the diffusion coefficient *D* and, for anisotropic materials, the diffusion tensor **D**^10,11^. The method is particularly powerful for studies of white matter; being widely used to correlate microstructural changes with diseases^12,13^, normal brain development^14,15^, and learning^16^.

With a volume of approximately 1 mm3, most imaging voxels contain multiple types of tissue with varying diffusion and relaxation properties^17,18^, all of which contributing to the measured MRI signal. Because of this heterogeneity, the signal from each voxel is more accurately described with distributions rather than unique values of the observables. Encoding of the MRI signal for information about positions, displacements, and relaxation requires tens to hundreds of milliseconds, a time during which the observables are averaged by exchange processes and micrometre-scale displacements. The underlying microstructure is captured by a distribution of effective observables that is related to the signal data via a Laplace transformation. Estimating a distribution from discretely sampled and noisy data by inverse Laplace transformation is a highly challenging problem in numerical analysis and in general requires regularization to improve the stability of the solution at the expense of introducing inversion artefacts^19–21^. Despite these difficulties, continuous *R*_2_-distributions have proven useful for mapping the fraction of myelin water^4,5^. Multidimensional Laplace NMR^22–27^ yields improved characterization of heterogeneous materials by estimating joint distributions of observables such as *R*_1_, *R*_2_, and *D*. In order to resolve a discrete component, it is sufficient that just one of the available observables has a value that is substantially different from the other components. Resolution in one of the dimensions allows for estimation of minor differences in the other observables that would be challenging to detect in the one-dimensional Laplace approach.

All multidimensional Laplace approaches to date have been limited to correlations between one or more relaxation rates and the scalar diffusion coefficient *D* rather than the diffusion tensor **D**. Diffusion encoding in a single direction convolves the effects of diffusion anisotropy and the orientation of the diffusion tensor eigenvectors, giving rise to *D*-distributions with complex shapes^28^ that are impossible to reproduce with regularized Laplace inversion because of the well-known artefacts. Neglecting this fact when applying multidimensional Laplace methods to heterogeneous anisotropic materials, such as brain tissue, will invariably produce distributions fraught with artefacts that, although being numerically stable, are more related to the choice of regularization than the sought-for structure of the investigated material. Alternatively, the number of the components, as well as some of their properties, can be assumed using prior knowledge of the underlying structure, leaving a few parameters to be estimated by fitting the model to the experimental data^29^. Within the typical signal-to-noise ratio (SNR) in MRI, the same data can often be equally well described with fundamentally different models^30,31^, or even distinct parameter sets for the same model^32,33^. The validity of the analysis is difficult to ascertain since the results depend just as much on the assumptions as on the data.

We have recently used principles from solid-state nuclear magnetic resonance (NMR) spectroscopy^34^ to design new diffusion MRI methods for investigating heterogeneous anisotropic materials^35–39^. The main insight from solid-state NMR is that the effects of diffusion tensor anisotropy and orientation can be disentangled by acquiring multidimensional data with correlations between isotropic and directional diffusion encoding^37,38^. Model-free and unconstrained inversion of the multidimensional data allows estimating a diffusion tensor distribution (DTD)^39–43^ that is directly related to the distribution of microscopic environments in the material. Here, we join the DTD and Laplace frameworks into a single experimental method that combines the simple relation between diffusion tensors and microstructure with the sensitivity of *R*_1_ and *R*_2_ to the chemical composition of the tissue^1–6^. In this novel approach, a heterogeneous sample is characterized through correlations between the isotropic diffusivity *D*_iso_, normalized diffusion anisotropy *D*_Δ_, orientation of the diffusion tensor (*θ*, *ϕ*), and relaxation rates *R*_1_ and *R*_2_.

The method at hand is tested with both simulations and experiments conducted on a phantom composed of an aligned liquid crystal and a yeast suspension, a schematic of which can be found elsewhere^37^. The used phantom mimics the diffusion properties of the nerve tissue model proposed by Stanisz *et al*.^29^; the liquid crystalline and intracellular yeast components resemble water within axons and glial cells respectively, while the extracellular environment of the yeast suspension replicates the diffusion properties of nerve tissue’s extracellular water. Spectroscopic proof-of-concept experiments were performed using Bruker microimaging hardware and a modified version of the NMR pulse sequence introduced in ref.^44^. While the experimental implementation presented here is too demanding for the hardware constraints of clinical MRI scanners, the proposed method can be redesigned using the smooth gradient waveforms from similar *in vivo* studies^40,45,46^. We consider this contribution as an important step towards the development of a protocol for the model-free quantification of the fractions of different structural components of the brain.

## Theory

Diffusion MRI techniques are sensitive to molecular displacements at the 10–100 ms time-scale. Such sensitivity allows us to probe the underlying microstructure of a biological tissue by measuring its effects on the translational motion of water. The measured displacements can be approximated by an apparent diffusion tensor **D** that depends on both the geometry of the pore space^38,47^ and the timing parameters of the used MRI sequence. Within the DTD model^39–43^, a heterogeneous material is pictured as a collection of independent microscopic domains whose structure is characterized by a corresponding microscopic **D**. Combining the DTD and Laplace NMR frameworks, the composition of a voxel can be characterized by the distribution *P*(**D**, *R*_1_, *R*_2_) which is mapped into the signal amplitude *S* by a kernel *K*(*τ*_R_, *τ*_e_, **b**, *R*_1_, *R*_2_, **D**) according to

1 $$S({\tau }_{{\rm{R}}},{\tau }_{{\rm{e}}},{\bf{b}})={S}_{0}{\int }_{0}^{\infty }{\int }_{0}^{\infty }{\int }_{0}^{\infty }P({R}_{1},{R}_{2},{\bf{D}})K({\tau }_{{\rm{R}}},{\tau }_{e},{\bf{b}},{R}_{1},{R}_{2},{\bf{D}})\,{\rm{d}}{R}_{1}\,{\rm{d}}{R}_{2}\,{\rm{d}}{\bf{D}},$$

where *τ*_R_ and *τ*_e_ are, respectively, the repetition and echo times, and **b** denotes the diffusion encoding tensor^40,48,49^, all of which being parameters under direct experimental control. *S*_0_ symbolizes the signal obtained when no diffusion or relaxation encoding is applied. The “biophysical modelling”^29–33^ approach of analysing MRI data can be seen as a post-acquisition procedure where a functional form of *P*(…) is assumed and fitted to the signal attenuation curve. Because the dimensionality and form of *K*(…) are controlled by the choice of pulse sequence and sampling scheme, the acquired data is intrinsically linked to the experimental protocol.

In typical relaxation and diffusion MRI protocols, an “encoding block”, wherein *R*_1_, *R*_2_, and *D* modulate the magnetization, precedes a block where the signal is read out. The modulation details are contained within an appropriate kernel, whose functional form is imprinted into the signal via equation (1). At the micrometre length scale, where relevant cellular features appear, the effects of relaxation and diffusion on the detectable transverse magnetization are well described by the Bloch-Torrey equation^50,51^. While the general solution of the Bloch equations is highly complex, a simple exact expression can be attained if one neglects the presence of boundaries and exchange phenomena^52,53^. Being commonplace in studies of biological tissues and heterogeneous materials^30–32,39–43^, such approximation is normally referred to as the Gaussian or free diffusion assumption. Its use yields the following kernel^22^:

2 $$K({\tau }_{{\rm{R}}},{\tau }_{e},{\bf{b}},{R}_{1},{R}_{2},{\bf{D}})=[1-\exp (-{\tau }_{{\rm{R}}}{R}_{1})]\exp (-{\tau }_{{\rm{e}}}{R}_{2})\exp (-{\bf{b}}:{\bf{D}}),$$

where **b**:**D** denotes a generalized scalar product defined as ${\sum }_{i}{\sum }_{j}{b}_{{ij}}{D}_{{ij}}$. The factors $[1-\exp (-{\tau }_{{\rm{R}}}{R}_{1})]$, $\exp (-{\tau }_{{\rm{e}}}{R}_{2})$, and $\exp (-{\bf{b}}:{\bf{D}})$ describe the weighting introduced by longitudinal recovery, transverse relaxation, and diffusion, respectively. The diffusion-encoding factor shows that **D** is measured relative to a *b*-tensor, which is implemented through a set of carefully chosen gradient waveforms. As demonstrated below via an explicit expansion of **b**:**D**, the parameterization of **b** can be manipulated to target specific diffusion features.

A symmetric positive definite second order tensor can be represented as a 3 × 3 matrix, and fully characterized by 6 independent elements^38–40^. Imposing an axial symmetry constraint, the number of independent values is reduced to four; two of them characterize the tensor orientation, and the other two define its size and shape parameters. In its principal axis system (PAS), an axial symmetric tensor **Λ** can be parameterized by its axial and radial eigenvalues ${\lambda }_{\parallel }$ and *λ*_⊥_, respectively. Alternatively, a complete description of **Λ** can be devised on the basis of its isotropic average *λ*_iso_ and normalized anisotropy *λ*_Δ_^38,39,48^:

3 $$\begin{array}{c}{\lambda }_{{\rm{iso}}}=\frac{\lambda }{3}=\frac{{\lambda }_{||}+2{\lambda }_{\perp }}{3},\\ {\lambda }_{{\rm{\Delta }}}=\frac{{\lambda }_{||}-{\lambda }_{\perp }}{3{\lambda }_{{\rm{iso}}}};\end{array}$$

where *λ* is the trace of **Λ**. While the relative “size” of distinct tensors are represented by their corresponding *λ*_iso_ values, *λ*_Δ_ reports on the “shape” of **Λ** with *λ*_Δ_ < 0, *λ*_Δ_ = 0, and *λ*_Δ_ > 0 describing oblate, spherical, and prolate tensors, respectively.

In the diffusion MRI literature, both **b** and **D** are usually approximated as axisymmetric second order tensors^30–32,39–43^ that can then be conveniently parameterized by the above metric. Following refs^35,38^:

4 $${\bf{D}}={D}_{{\rm{iso}}}\{{\bf{I}}+{D}_{{\rm{\Delta }}}[\begin{array}{ccc}3{l}_{x}^{2}-1 & 3{l}_{x}{l}_{y} & 3{l}_{x}{l}_{z}\\ 3{l}_{x}{l}_{y} & 3{l}_{y}^{2}-1 & 3{l}_{y}{l}_{z}\\ 3{l}_{x}{l}_{z} & 3{l}_{y}{l}_{z} & 3{l}_{z}^{2}-1\end{array}]\},$$

where **I** is the identity matrix, and

5 $$\begin{array}{rcl}{l}_{x} & = & \cos \,\varphi \,\sin \,\theta ,\\ {l}_{y} & = & \sin \,\varphi \,\sin \,\theta ,\\ {l}_{z} & = & \cos \,\theta ;\end{array}$$

with *θ* and *ϕ* denoting the polar and azimuthal angles that define the orientation of the *D*-tensor PAS in respect to the laboratory frame of reference. Using a similar parameterization for **b**, we can write^48,54^:

6 $${\bf{b}}=\frac{b}{3}\{{\bf{I}}+{b}_{{\rm{\Delta }}}[\begin{array}{ccc}3{l}_{x}^{2}-1 & 3{l}_{x}{l}_{y} & 3{l}_{x}{l}_{z}\\ 3{l}_{x}{l}_{y} & 3{l}_{y}^{2}-1 & 3{l}_{y}{l}_{z}\\ 3{l}_{x}{l}_{z} & 3{l}_{y}{l}_{z} & 3{l}_{z}^{2}-1\end{array}]\},$$

where *b* corresponds to the trace of the *b*-tensor, and *b*_Δ_ is its normalized anisotropy. The *l*_*i*_ quantities are defined through equation (5), with the (*θ*, *ϕ*) variables replaced by (Θ, Φ), the polar and azimuthal angles describing a rotation of **b** through the laboratory frame.

Combining equations (4) and (6), the double scalar product of equation (2) can be expanded as

7 $${\bf{b}}:{\bf{D}}=b{D}_{{\rm{iso}}}[1+2\,{b}_{{\rm{\Delta }}}{D}_{{\rm{\Delta }}}{P}_{2}(\cos \,{\rm{\Theta }}\,\cos \,\theta +\,\cos ({\rm{\Phi }}-\varphi )\sin \,{\rm{\Theta }}\,\sin \,\theta )]$$

where *P*_2_(*x*) = (3*x*^2^ − 1)/2 is the 2^nd^ Legendre polynomial, and its argument can be rewritten as the cosine of the arc-angle *β* between the *b*- and diffusion tensors. Equation (7) highlights the effects of the *b*-tensor shape on the measured signal decay. A linear *b*-tensor (*b*_Δ_ = 1) returns an effective diffusion coefficient *D*_eff_ = *bD*_iso_[1 + 2*D*_Δ_*P*_2_(*β*)] defined by the size, anisotropy, and orientation of **D**, whereas spherical diffusion encoding (*b*_Δ_ = 0) averages out the effects of anisotropy and orientation, thus allowing the direct measurement of *D*_iso_.

Insertion of equations (7) and (2) into (1) yields

8 $$\begin{array}{c}S({\tau }_{{\rm{R}}},{\tau }_{{\rm{e}}},b\,,{b}_{{\rm{\Delta }}},{\rm{\Theta }},{\rm{\Phi }})={S}_{0}{\int }_{0}^{\infty }{\int }_{0}^{\infty }{\int }_{0}^{\infty }{\int }_{-1/2}^{1}{\int }_{0}^{\pi }{\int }_{0}^{2\pi }K({\tau }_{{\rm{R}}},{\tau }_{{\rm{e}}},b\,,{b}_{{\rm{\Delta }}},{\rm{\Theta }},{\rm{\Phi }},{R}_{1},{R}_{2},{D}_{{\rm{iso}}},{D}_{{\rm{\Delta }}},\theta ,\varphi )\\ \,\,\,\,\,\,\,\,\times P\,({R}_{1},{R}_{2},{D}_{{\rm{iso}}},{D}_{{\rm{\Delta }}},\theta ,\varphi ){\rm{d}}\varphi \,\sin \,\theta \,{\rm{d}}\theta \,\,\,{\rm{d}}{D}_{{\rm{\Delta }}}{\rm{d}}{D}_{{\rm{iso}}}\,\,{\rm{d}}{R}_{2}{\rm{d}}{R}_{1}\,,\end{array}$$

wherein size, anisotropy and orientation of the tensors are written as separate dimensions of the acquisition, (*τ*_R_, *τ*_e_, *b*, *b*_Δ_, Θ, Φ), and analysis, (*R*_1_, *R*_2_, *D*_iso_, *D*_Δ_, *θ*, *ϕ*), spaces. Equation (8) belongs to a class of integral transforms whose inversion is notoriously difficult, with an infinite number of distinct distributions *P*(*R*_1_, *R*_2_, **D**) being consistent with the experimental signal^55^. To perform an unconstrained estimation of the assumedly sparse distribution *P*(*R*_1_, *R*_2_, *D*_iso_, *D*_Δ_, *θ*, *ϕ*), enough information has to be encoded into the multidimensional signal in order to render *S*(*τ*_R_, *τ*_e_, *b*, *b*_Δ_, Θ, Φ) specific to the sample’s microstructure. However, the high dimensionality of our acquisition space complicates the task of comprehensively sampling the (*τ*_R_, *τ*_e_, *b*, *b*_Δ_, Θ, Φ) set within a reasonable experimental time. Traditional Laplace NMR sampling schemes consist of rectangular grids wherein the amount of sampling is given by *n*_1_ × *n*_2_ × … × *n*_*i*_, with *n*_*i*_ being the number of experimental points in the *i*-th dimension of the acquisition space. In our 6D space, the use of a grid scheme would then allow very low *n*_*i*_ before the total number of samples becomes prohibitively high.

**Figure 1:**
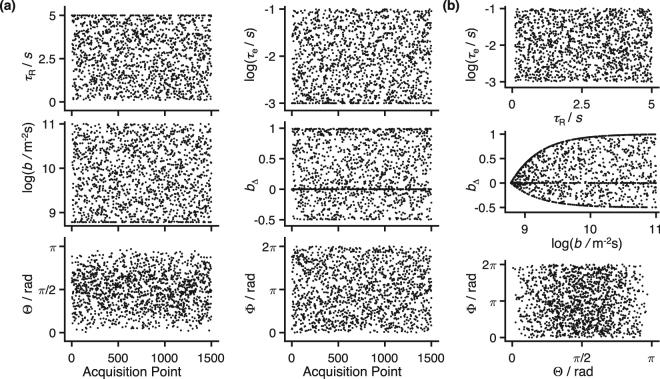
Pseudo-random data acquisition protocol capable of establishing correlations between the six different dimensions of the (*R*_1_, *R*_2_, *D*_iso_, *D*_Δ_, *θ*, *ϕ*) space. (**a**) The black dots denote the various (*τ*_R_, *τ*_e_, *b*, *b*_Δ_, Θ, Φ) space coordinates, sampled at distinct acquisition points. (**b**) Selected 2D projections of the original six-dimensional acquisition space. The non-uniform density observed in some of the plots is the result of a sampling bias introduced and controlled by the experimentalist.

To circumvent the aforementioned problem we opt to follow the pseudo-random strategy shown in Fig. 1, which establishes 6D correlations across the (*R*_1_, *R*_2_, *D*_iso_, *D*_Δ_, *θ*, *ϕ*) space by randomly selecting combinations of (*τ*_R_, *τ*_e_, *b*, *b*_Δ_, Θ, Φ). Compared to the grid design, the pseudo-random approach allows a more thorough sampling of each element of (*τ*_R_, *τ*_e_, *b*, *b*_Δ_, Θ, Φ) for the same total number of acquired data points. The increase of sampling density per dimension, coupled to the introduction of a bias towards higher signal values, increases the sensitivity of *S*(*τ*_R_, *τ*_e_, *b*, *b*_Δ_, Θ, Φ) to the analysed material. The specificity of the acquired data, coupled with the assumption of a sparse analysis space, allows the model-free determination of *P*(*R*_1_, *R*_2_, *D*_iso_, *D*_Δ_, *θ*, *ϕ*) via an appropriate inversion algorithm^21,56–58^. A comparison between the pseudo-random scheme and a grid-like sampling scheme is presented in the Supplementary Information. While the proposed acquisition scheme is reminiscent of the non-uniform sampling protocols from multidimensional NMR spectroscopy^59,60^ or MRI fingerprinting^61^, the data analysis of this contribution follows a different methodology than the one of the cited works.

Before progressing any further, one should discuss the validity of our signal model. The derivation of Eq. (8) rests upon the two base assumptions of the DTD model: diffusion inside the different micro-domains is Gaussian and exchange of diffusing particles between the various domains is non-existent. When those assumptions are not met, the protocol measures an effective set of (*R*_1_, *R*_2_, *D*_iso_, *D*_Δ_, *θ*, *ϕ*)-values that are averaged by the water’s residence time in the different domains and are highly dependent on the choice of experimental time parameters. Furthermore, neglecting the effects of restricted diffusion may result in the estimation of different apparent diffusivities for distinct values of *b*_Δ_ (see Supplemental Material of ref.^37^) or a bias in the estimation of domain sizes^62^. However, despite its limitations, the DTD model has shown to be a useful departure point for MRI studies of the human brain^40^.

**Figure 2:**
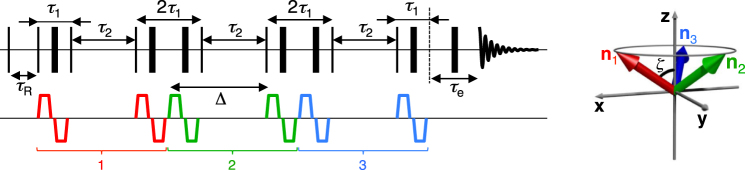
Pulse sequence for encoding diffusion tensor **D** and nuclear relaxation effects. The thin vertical lines denote 90°_*x*_ RF pulse while the thick vertical lines represent 180°_*y*_ pulses. The sequence relies on the detection of a stimulated echo, whose magnitude is encoded for longitudinal recovery *R*_1_, transverse relaxation *R*_2_ and **D** in three individual blocks. Relaxation encoding is done through the variation of the *τ*_R_ and *τ*_e_ delays, which weight the effects of *R*_1_ and *R*_2_, respectively. The signal is further affected by *R*_2_ and *R*_1_ during the constant *τ*_1_ and *τ*_2_ delays, respectively. Diffusion is modulated by three sets of bipolar gradient pulses whose unit vectors **n**_1_, **n**_2_, and **n**_3_ are shown in the right panel. The colour-code of the direction vectors is consistent with that of the pulse sequence brackets, showing that (**n**_1_, **n**_2_, **n**_3_) have a three-fold azimuthal symmetry around an axis over which they hold a constant polar angle *ζ*. The *b*-tensor anisotropy is tuned by changing *ζ*, as described by Eq. (9). (Adapted from ref.^44^. Copyright 2015 by Elsevier.)

A pulse sequence capable of encoding the NMR signal for both diffusion and nuclear relaxation is displayed in Fig. 2. The sequence is based on a previous triple-stimulated echo protocol^44^, to which an initial 90° pulse and subsequent recovery time were added in order to grant access to the full 6D acquisition space. Variation of *τ*_R_ and *τ*_e_ allows signal modulation by *R*_1_ and *R*_2_. Diffusion encoding is performed by three consecutive pairs of bipolar gradient pulses, whose direction vectors **n**_1_, **n**_2_, and **n**_3_ are equally distributed on the surface of a cone of aperture 2*ζ*. The angle *ζ* is directly related to the anisotropy of **b** through^44^

9 $${b}_{{\rm{\Delta }}}={P}_{2}(\cos \,\zeta ).$$

A continuous sampling of axisymmetric *b*-tensor “shapes” can be performed by varying *ζ*; from linear diffusion-weighting at *ζ* = 0°, via spherical encoding when *ζ* = acos(1/3^1/2^) ≈ 54.74°, to planar diffusion weighting at *ζ* = 90°.

## Results

**Figure 3:**
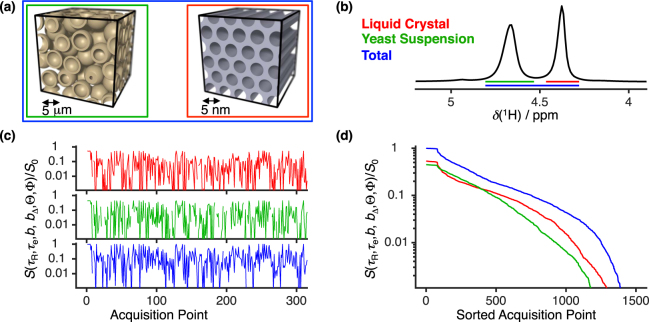
Link between structure and raw experimental data. (**a**) Illustration of the phantom’s microscopic geometry. The yeast suspension microstructure (green frame) is pictured as a set of spherical shells representing the cell membranes that delimit the intra- and extracellular domains. A liquid crystal microdomain (red frame) is seen as a coherent set of cylindrical water channels in a continuous matrix of detergent and hydrocarbon. The blue frame demarks the entire sample volume. (**b**) ^1^H NMR spectrum displaying the separate water chemical shifts from the liquid crystalline solution, and the yeast suspension. The coloured bars beneath the spectrum follow the same colour-code as the frames in (**a**), and identify the various contributions to the spectrum. (**c**) Signal evolution registered during the first 300 acquisition points. (**d**) Decay curves displaying the acquired data sorted in the order of descending signal amplitudes. The colour scheme of (**c**) and (**d**) matches the chemical-shift resolved decays with the integrated spectral regions.

For the proof-of-concept experiments we used the NMR pulse sequence in Fig. 2 to acquire 1500 experimental points sampled according to the scheme of Fig. 1. The water resonance lines from the liquid crystal and the yeast suspension are narrow enough to be resolved in the chemical shift dimension (Fig. 3a). Hence, the signal decays from the sample’s two constituents can either be separated or merged depending on which spectral regions are integrated. Figure 3c and d show that samples with distinct microstructure give rise to different signal patterns, thus highlighting that the signal pattern is specific to the underlying *P*(*R*_1_, *R*_2_, *D*_iso_, *D*_Δ_, *θ*, *ϕ*) function.

**Figure 4:**
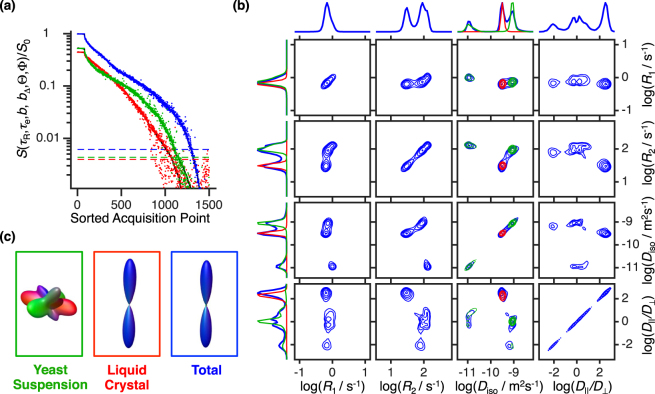
Six dimensional *P*(*R*_1_, *R*_2_, *D*_iso_, *D*_||_*/D*_⊥_, *θ*, *ϕ*) distribution calculated through unconstrained inversion of data acquired with the phantom. The colours follow the same code of Fig. 3; red and green respectively denote the inversion results for the liquid crystal and yeast components, and blue represents the results for the entire sample volume. (**a**) Water signal decays for different volume sets of the used phantom. The solid black lines identify the experimental signal, the coloured points represent the fitting results, and the dashed coloured lines represent the noise level estimated from the fit’s residuals. (**b**) Projections of the full *P*(*R*_1_, *R*_2_, *D*_iso_, *D*_||_*/D*_⊥_, *θ*, *ϕ*) probability spectrum onto 2D and 1D subsets of (*R*_1_, *R*_2_, *D*_iso_, *D*_||_*/D*_⊥_). The 4 × 4 contour plot set displays all the 2D distributions contained within the (*R*_1_, *R*_2_, *D*_iso_, *D*_||_*/D*_⊥_) space, the individual dimensions of which are projected on the top and left side of the set. The contour lines are linearly spaced from 7.5% to 90% of the maximum value. (**c**) Three dimensional surface plot representing the orientation distribution *P*(*θ*, *ϕ*) for domains where *D*_||_*/D*_⊥_ > 10. While the frames identify the sample’s sub-volumes (green = yeast suspension; red = liquid crystal; blue = yeast + liquid crystal), the 3D plot itself is color-coded as [red, green, blue] = [cos*ϕ*sin*θ*, sin*ϕ*sin*θ*, cos*θ*]. Since the yeast sample contains no anisotropic domains, the orientation distribution function recovered for that component consists of numerical noise originated by the *D*_||_*/D*_⊥_ spread of the intra-cellular population.

Capitalizing on the specificity of our acquisition protocol, the distribution *P*(*R*_1_, *R*_2_, *D*_iso_, *D*_Δ_, *θ*, *ϕ*) was retrieved from the measured data through a model-free inversion of Eq. (8). As elaborated upon in the Supplementary Information, the inversion procedure followed the same approach of our previous correlation works^37,39^. Briefly, Eq. (8) is inverted through a non-negative least squares fitting algorithm augmented with bootstrap^63^ resampling. Our procedure allows the estimation of an ensemble of equally valid *P*(*R*_1_, *R*_2_, *D*_iso_, *D*_Δ_, *θ*, *ϕ*) solutions, the average of which is reported in Fig. 4 as a set of contour maps. Besides the non-negative constraint and a restriction on the range of allowed distributions, no further regularization constraints were used.

The probability spectrum displayed in Fig. 4 resolves the three microscopic components of the composite sample on a (*R*_1_, *R*_2_, *D*_iso_, *D*_||_*/D*_⊥_ = (1 + 2*D*_Δ_)/(1−*D*_Δ_), *θ*, *ϕ*) basis. The choice to parameterize anisotropy with *D*_||_*/D*_⊥_ is justified by the fact that, unlike the *D*_Δ_ parameter, *D*_||_*/D*_⊥_ > 0 for any given “shape” and can thus be represented in a logarithmic scale. Yielding similar relaxation rates (log(*R*_1_/s^−1^) ≈ 0 and log(*R*_2_/s^−1^) ≈ 2), the intra- and extracellular yeast compartments are separated as two isotropic components with log(*D*_iso_/m^2^s^−1^) ≈ −9 and −11, respectively. The measurement of a relaxation time *T*_1_ = 1/*R*_1_ longer than the intracellular lifetime of yeast, *τ* ≈ 0.5 s^64^, indicates that the *R*_1_ values obtained for both intra- and extracellular water are partially averaged by molecular exchange. The anisotropic population at [log(*R*_1_/s^−1^), log(*R*_2_/s^−1^), log(*D*_iso_/m^2^s^−1^), log(*D*_||_*/D*_⊥_)] ≈ (0, 1.5, −9, 2) originates from the liquid crystal. The orientation distribution function *P*(*θ*, *ϕ*) of the anisotropic component is naturally recovered and is represented as a 3D surface plot in Fig. 4c. Its shape and color-coding are consistent with an alignment along the *z*-direction of the laboratory frame, which coincides with the main magnetic field.

**Figure 5:**
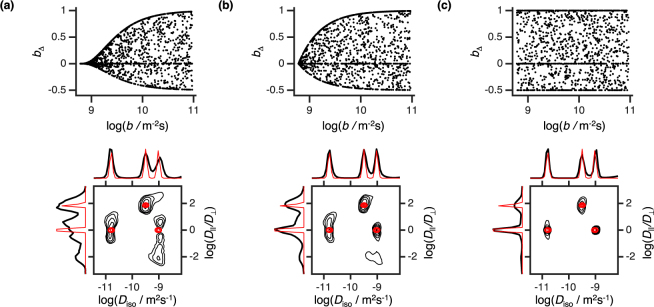
Inversion of data simulated with different acquisition biases. Columns (**a**–**c**) show the effects of different *b*_Δ_ sampling schemes on the estimated 6D probability function. The sampling scheme of the (*τ*_R_, *τ*_e_, *b*, Θ, Φ) coordinates, the SNR, and the number of acquisition points were all kept constant across different columns. The scatter plots on top of each column show the specific bias introduced on the (*b*, *b*_Δ_) projection and the black contour plots show the 2D *P*(*D*_iso_, *D*_||_*/D*_⊥_) projections of the resulting probability distribution. The ground-truth is overlain in red. Column (**b**) shows data simulated with the sampling scheme of Fig. 1. Columns (**a**) and (**c**) show the effects of reducing or increasing, respectively, the range of *b*_Δ_ at low *b*-values.

Besides the three expected components, the (*D*_iso_, *D*_||_*/D*_⊥_) map also displays an oblate population with the same *R*_1_, *R*_2_, and *D*_iso_ values as the fast isotropic component. Inconsistent with the phantom’s design, the existence of this component can be attributed to an inversion artefact resulting from the under-sampling of *b*_Δ_ at low *b*-values (see second plot of Fig. 1b). Since not enough “shape” information was encoded into the initial decay, the noise fluctuations accommodate an extra anisotropic component that bears no physical meaning. As demonstrated in Fig. 5, the presence of the oblate population can be corrected by increasing the *b*_Δ_ range at lower *b*-values. While such modifications could be implemented without increasing the sampling density, we choose to keep such artefact for illustrative purposes. The spread of isotropic components over the *D*_||_*/D*_⊥_ axis offers an additional example of artefacts resultant from the data noise^65,66^, the presence of which clearly shapes the correlation map without decreasing the fit quality (See Supplementary Figure S1).

**Figure 6:**
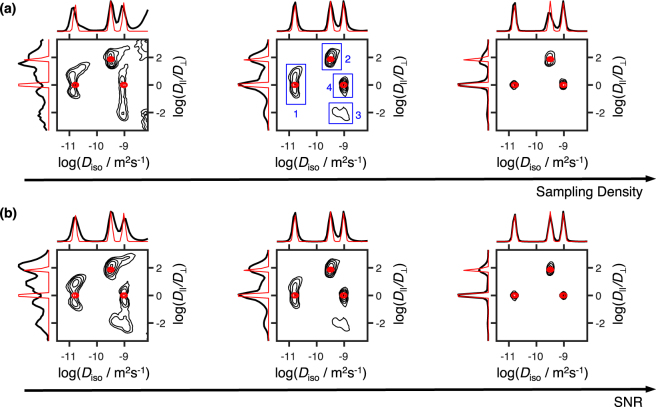
Simulations showing the effects of sampling density and SNR on the model-free estimation of *P*(*R*_1_, *R*_2_, *D*_iso_, *D*_||_*/D*_⊥_, *θ*, *ϕ*). The various contour plots show the 2D projections of the ground-truth (red line) and the full inverted distribution (black line) onto the (*D*_iso_, *D*_||_*/D*_⊥_) space. (**a**) Contour plots showing the inversion of data simulated with varying number of acquisition points (from left to right: 500 points, 1500 points, 5000 points) and a constant SNR of 120. (**b**) Distributions estimated for various levels of SNR (from left to right: SNR = 60, SNR = 120, SNR = 400) and a constant sample size of 1500 points. The blue numbered boxes identify the four water populations observed in the experimental dataset of Fig. 4.

The limits of our method were further assessed with data simulated for a discrete three-compartment voxel compatible with the used phantom. The artificial signal was generated using the pseudo-random acquisition protocol displayed in Fig. 1 and the (*τ*_R_, *τ*_e_, *b*, *b*_Δ_, Θ, Φ) set described in the *Methods* section. Gaussian distributed noise with an amplitude of 1/SNR was added to the data. Figure 6 shows the (*D*_iso_, *D*_Δ_) maps estimated from the model free inversion of signals simulated using different sampling densities and SNR values. Although the entire *P*(*R*_1_, *R*_2_, *D*_iso_, *D*_Δ_, *θ*, *ϕ*) distribution was inverted, we only display the *P*(*D*_iso_, *D*_Δ_) projection to facilitate the interpretation of the figure. The overall properties of the *P*(*R*_1_, *R*_2_, *D*_iso_, *D*_Δ_, *θ*, *ϕ*) recovered from experimental data can be see in simulations with the same sample size and an intermediate SNR. As expected, the inversion quality increases with increasing sample size and SNR as the amount of information encoded in the signal directly correlates with those two properties.

**Figure 7:**
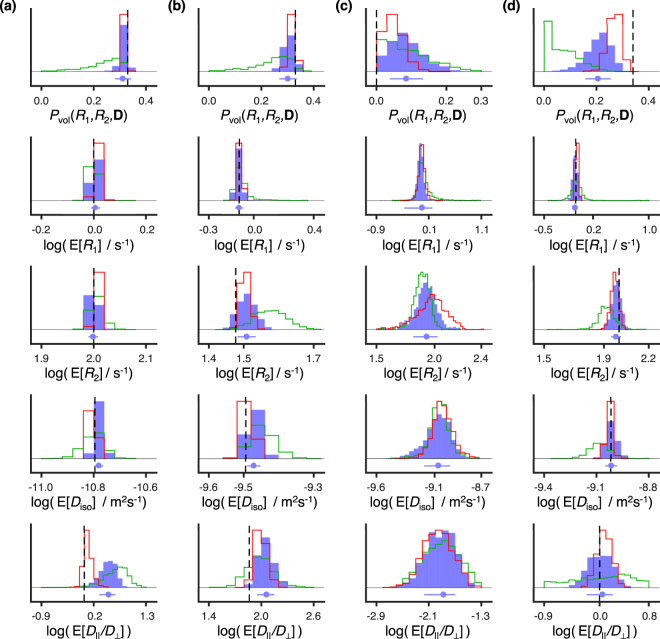
Bootstrap averages of the weight, *R*_1_, *R*_2_, *D*_iso_, and *D*_||_*/D*_⊥_ of four distinct microscopic environments. The histograms were computed from 1000 solutions of Eq. (8) for the simulated data displayed in Fig. 6. The mean values, E[*X*], were calculated per bootstrap sample, for each of the four sub-volumes in Fig. 6. The various colours showcase values estimated from artificial datasets with different sampling densities (green line = 500 points; blue boxes = 1500 points; red line = 5000 points). The black dotted line displays the ground truth. Columns (**a**), (**b**), (**c**), and (**d**) contain the statistics of the components demarked by rectangles 1, 2, 3, and 4, respectively, in Fig. 6. The blue dot and line underneath the histogram bars indicate the mean and standard deviation, respectively, of the bootstrap distributions from the dataset with 1500 points. Histograms of a given variable (*P*_vol_(*R*_1_, *R*_2_, **D**), E[*R*_1_], E[*R*_2_], E[*D*_iso_], or E[*D*_||_*/D*_⊥_]) have a constant bin width across the various columns.

In the spirit of Prange and Song^66,67^, we used the variability between solutions to devise histograms showing the intrinsic variability of the resolved populations (see Fig. 7). The statistical analysis was carried out for four different sub-volumes of our analysis space, each of them containing one of the various components identified in Fig. 6. Focusing on the distribution of the weight of each sub-volume, *P*_vol_(*R*_1_, *R*_2_, *D*_iso_, *D*_Δ_, *θ*, *ϕ*), one notices that regions comprising higher diffusivities (Fig. 7c and d) carry a higher uncertainty. Such behaviour can be interpreted as a symptom of the “shape” undersampling at low *b* and is at the origin of the oblate artefact, which disappears for higher sampling densities. While the solutions from the 500 points dataset completely fail to resolve the various populations, both 1500 and 5000 points are shown to return average values close to the underlying ground-truth. In fact, despite the presence of an artefact component, the average values estimated from the 1500 points dataset are close to or within a standard deviation from the “true” distribution. The exception lies on the calculated *D*_Δ_ values, whose higher uncertainty lies in the non-trivial interdependence of this variable with the *D*_iso_-, *θ-*, and *ϕ*- dimensions.

## Discussion

The link between the dimensionality of the correlation space and resolution power is well established, being discussed in various multidimensional NMR reviews^23–25^. Recently, we have also shown that the projection of **D** unto a sparse basis of (*D*_iso_, *D*_Δ_, *θ*, *ϕ*) allows us to resolve details that would otherwise be concealed by broad distributions of *D*_eff_ wherein size, shape and orientation are intrinsically entangled^37–39^. Here, high dimensionality and sparsity are combined to endow the presented protocol with a much superior resolution power when compared to classical Laplace NMR methods, which build correlations in the non-sparse space of *D*_eff_ rather than probing the separate components of the full diffusion tensor. Focusing on the correlation maps and their respective projections in Fig. 4b we notice that the three components cannot be resolved in the 1D *D*_||_*/D*_⊥_-, *R*_1_- and *R*_2_- dimensions. Since the various microenvironments of our colloidal phantom possess very similar *R*_1_ and *R*_2_ rates, these dimensions do not significantly contribute to the resolution of the different components. The components of this particular phantom are only resolved in the *D*_iso_, *D*_||_*/D*_⊥_, and (*θ*, *ϕ*) dimensions, which are separated in our novel acquisition scheme. Besides aiding in the separation of water components, the information gained by separating the various eigenvalues of **D** allows the detection of subtle differences in *R*_1_- and *R*_2_- values that would not be visible in standard 1D or 2D relaxation correlation experiments. For example, the 2D projection *P*(*R*_2_, *D*_iso_) reports a slightly higher value of *R*_2_ for the intracellular yeast domain compared to the extracellular one, a behaviour consistent with previous findings^25,68^.

The proposed method can be schematized into three separate steps: pulse sequence, acquisition protocol, and inversion algorithm, all of which can be independently matched to the experimental conditions at hand. For instance, the pulse sequence in Fig. 2, which is useful in the study of materials with high values of *R*_2_, can be substituted by a gradient modulation more compliant with the hardware constraints of clinical scanners. The family of continuous gradient waveform methods recently used for *in vivo* diffusion MRI studies^40,45,46,69,70^ offers a suitable alternative. Similarly, the pseudo-random scheme could be replaced by a rectangular sampling grid, or *P*(*R*_1_, *R*_2_, *D*_iso_, *D*_Δ_, *θ*, *ϕ*) be retrieved through an inversion algorithm exploiting one of the many available regularization metrics^20,21,71,72^. The 6D correlation approach is also a natural template for the design of lower dimensional correlation experiments. For example, the experiments introduced in refs^37,39^ can be respectively seen as projections onto the 4D (*D*_iso_, *D*_Δ_, *θ*, *ϕ*) and 2D (*D*_iso_, *D*_Δ_) spaces. Just as well, any other projection space can be chosen, *e*.*g*. (*R*_1_, *R*_2_, *D*_iso_), albeit at a cost of resolution.

Being an inherently ill-posed mathematical problem, the inversion of Eq. (8) accommodates an ensemble of solutions that are compatible with *S*(*τ*_R_, *τ*_e_, *b*, *b*_Δ_, Θ, Φ) within the measured SNR^63,66,67^. In order to circumvent the inversion’s non-unique character, the solution space is typically constrained through the introduction of regularization. The most standard constraints are the consideration of a non-negative distribution function, the restriction of the analysis space to physically realistic ranges, and the introduction of a bias towards smooth distributions^58^. Although capable of mitigating the influence of noise or reducing over-fitting, common regularization strategies yield well-known artefacts that affect the shape of the retrieved distribution^22,25,58^. Hence, the solution’s non-uniqueness and prevalence of artefacts should always be kept in mind when designing or using such experiments, a fact overlooked in contemporary MRI literature^73^. In an attempt to explicitly show the non-unique character, we use a bootstrapping procedure to estimate hundreds of possible solutions, and display a select number of them in the Supplementary Information. Examples of inversion artefacts were singled out from Fig. 4 in the Results section. The existence of those artefacts can motivate a more precise strategy for the determination and quantification of the different domains. For example, the signal could be fitted to a discrete model, constructed according to any prior knowledge about the studied material, and whose number of components and constraints should be compatible with the model-free results. In the present case, that means we do not expect oblate domains. Alternatively, one could introduce regularization constraints penalizing the appearance of additional peaks^20,21,71,72^ or use a refined version of our inversion where all components are constrained to either isotropic or prolate geometries.

In summary, we have suggested and demonstrated an NMR protocol capable of characterizing a sample’s heterogeneity on the basis of the observables defined by the Bloch-Torrey equations. Through an exhaustive sampling of a 6D acquisition space, the full *P*(*R*_1_, *R*_2_, *D*_iso_, *D*_Δ_, *θ*, *ϕ*) distribution is accessed in a model-free fashion. Since the typical MRI voxel comprises multiple microscopic domains with varying chemical and diffusion properties, the presented method shows great potential for *in vivo* studies of the human brain. In particular, through the imposition of physiologically reasonable constraints, we expect that our protocol can serve as a basis for experiments capable of determining the composition of a voxel in terms of tissue and cell types.

## Methods

### Phantom Preparation

The liquid crystal preparation followed the recipe described in a recent publication^74^, having the composition 41.94 wt% water (Milli-Q quality), 13.94 wt% of the hydrocarbon 2, 2, 4-trimethylpentane (Sigma-Aldrich, Sweden), and 44.12 wt% of the detergent sodium 1, 4-bis(2-ethylhexoxy)-1, 4-dioxobutane-2-sulfonate (trade name AOT from Sigma-Aldrich, Sweden). At room temperature, the liquid crystal is in a reverse 2D hexagonal phase wherein water diffuses along cylindrical channels with ~5 nm diameter which span over lengths of hundreds of micrometres, thus giving rise to highly anisotropic diffusion^74^. The sample was heated to 25 °C, and left in the NMR magnet over the weekend in order to align the crystallites parallel to the spectrometer’s magnetic field^75,76^.

A cube of fresh baker’s yeast (Kronjäst AB) was acquired at a local supermarket, and mixed with tap water at a 1:1 volume ratio. The mixture was kept in its original container, and stored for 24 hours at room temperature in order to enable the sedimentation of yeast. The sediment was transferred to a 10 mm NMR tube by a syringe equipped with a 1 mm diameter needle. The resulting yeast suspension is composed of microscopic spherical cells with water populations in the intra- and extra-cellular domains. Both populations exhibit isotropic diffusion, but with a few orders of magnitude difference in apparent diffusivity^35,77^.

The flame sealed 5 mm tube containing the liquid crystal was inserted into the 10 mm tube containing the yeast cell suspension, with the larger tube being successively sealed with Parafilm tape. The composite phantom was centrifuged for 10 minutes at 223 *g* to create a denser packing of yeast cells at the bottom of the wider tube. Before starting the NMR measurements, the sample was placed inside the NMR spectrometer and left to equilibrate for 30 minutes at 18 °C.

### NMR Measurements

The experiments were conducted on a Bruker Avance-II 500 MHz spectrometer equipped with a MIC-5 probe fitted with a 10 mm RF insert. The temperature was kept at a constant value of 18 °C throughout the entire experiment time of 161 minutes. Measurements were performed with the pulse sequence of Fig. 2, using a delay *τ*_1_ of 6.4 ms, a longitudinal storage time *τ*_2_ equal to 64 ms, and a recycle delay of 0.1 s. Diffusion encoding was performed by symmetric trapezoidal gradients with a ramp time of 0.2 ms, a plateau time of 1.9 ms, and a peak amplitude of 0.6 T/m. The *τ*_R_ time interval is preceded by a set of three saturation pulses designed to null the longitudinal magnetization. Coherence pathway selection was performed with a two-step RF pulse and receiver phase cycle coupled with extensive use of spoiler gradients^44^.

A total of 1500 points were sampled according to the scheme of Fig. 1. On a first step, the measurement points were randomly selected from the subset of the (*τ*_R_, log(*τ*_e_), log(*b*), *b*_Δ_, cosΘ, Φ) space defined by the limits 0.1 < *τ*_R_/s < 5, 1 < *τ*_e_/ms < 100, 6.06·10^8^ < *b*/m^−2^s < 10^11^, −0.5 < *b*_Δ_ < 1, −1 < cosΘ < 1, and 0 < Φ/rad < 2π. Following random selection, *b*_Δ_ was resampled as *b*_Δ_ = *b*_Δ_·(*b* − 6.06·10^8^)/*b* in order to achieve sufficient spoiling effect of the gradient pulses even at low *b*-values^44^. Finally, 30%, 15%, 10%, and 5% biases towards the respective [*b*_Δ_ = 1–6.06·10^8^ m^−2^s/*b*], [*b*_Δ_ = 0], [*b*_Δ_ = −0.5–6.06·10^8^ m^−2^s/2*b*], and [*τ*_R_ = 5 s, *τ*_e_ = 10^−3^ s, *b* = 6.06·10^8^ m^−2^s] parameter sets were consecutively enforced into the acquisition space. The *b*_Δ_ = 1–6.06·10^8^ m^−2^s/*b*, *b*_Δ_ = 0, and *b*_Δ_ = −0.5–6.06·10^8^ m^−2^s/2*b* lines were oversampled as they contain the acquisition points wherein the signal is more sensitive to variations in *D*_Δ_^48^. The bias towards the [*τ*_R_ = 5 s, *τ*_e_ = 10^−3^ s, *b* = 6.06·10^8^ m^−2^s] parameter set was used to infer the stability of our experimental setup. The initial plateau observed in Fig. 3d shows that the experimental conditions were constant throughout our measurement time, with no evidence of signal drifts being observed^78^.

All data processing was carried out with in-house code written in Matlab (The Mathworks, Natick, MA). A more detailed account on the data inversion algorithm can be found in the Supplementary Information.

### Data availability

The dataset described in the current study are available from the corresponding author on reasonable request. MATLAB code for the data inversion is freely available at https://github.com/daniel-topgaard/md-dmri.

## Electronic supplementary material

Supplementary Information
