# Supplementary material for: Multidimensional correlation of nuclear relaxation rates and diffusion tensors for model-free investigations of heterogeneous anisotropic porous materials
Source: Sci Rep. 2018 Feb 6;8:2488. doi: 10.1038/s41598-018-19826-9 (PMC5802831; doi:10.1038/s41598-018-19826-9)
Supplement: Supplementary file 1 — Supplementary Information [file 41598_2018_19826_MOESM1_ESM.pdf]

# Multidimensional correlation of nuclear relaxation rates and diffusion tensors for model-free investigations of heterogeneous anisotropic porous materials

*João P. de Almeida Martins*[1,*], *Daniel Topgaard*[1]

[1]*Division of Physical Chemistry, Department of Chemistry, Lund University, Lund, Sweden*
**Corresponding author: joao.martins@fkem1.lu.se*

## NUMERICAL INVERSION

Eq. (8) from the main text was inverted via the numerical algorithm previously used in references 1 and 2, whose implementation on MATLAB is freely available at https://github.com/daniel-topgaard/md-dmri. The continuous distribution $P(R_1,R_2,D_{\mathrm{iso}},D_\Delta,\theta,\phi)$ is rewritten as an $N\times1$ (rows×columns) vector $\mathbf{w}$ whose elements $w_\mathrm{n}$ weight $N$ different nodes of a discretized $(R_1,R_2,D_{\mathrm{iso}},D_\Delta,\theta,\phi)$ space. Based on early non-negative least squares (NNLS) approaches[3], $\mathbf{w}$ is retrieved through an iterative minimization of the expression

$$\left\|\mathbf{Kw}-\mathbf{s}\right\|^2, \tag{S1}$$

where ||•|| denotes the $L_2$-norm, $\mathbf{s}$ is a $M\times1$ vector of signal amplitudes acquired for $M$ different points of the $(\tau_\mathrm{R},\tau_\mathrm{e},b,b_\Delta,\Theta,\Phi)$ space, and $\mathbf{K}$ a $M\times N$ matrix whose entries are given by Eq. (2) of the main text. Here, we only provide a brief description of our numerical approach; readers interested in additional details are referred to the documentation on GitHub. The used MATLAB code can be summarized in 6 steps:

1. A "parent" basis, $(R_1,R_2,D_\|,D_\perp,\theta,\phi)_\mathrm{P}$, is generated through random selection of 500 nodes from the $(\log(R_1),\log(R_2),\log(D_\|), \log(D_\perp), \cos\theta, \phi)$ space;
2. Eq. (S1) is minimized using MATLAB's standard NNLS algorithm in order to estimate the weights $w_\mathrm{n}$ for the different members of the basis. The basis together with its corresponding $\mathbf{w}$ define a solution;
3. A new "offspring" basis, $(R_1,R_2,D_\|,D_\perp,\theta,\phi)_\mathrm{O}$, is generated by adding a small perturbation to the $(R_1,R_2,D_\|,D_\perp,\theta,\phi)_\mathrm{P}$ basis. Mathematically: $(R_1,R_2,D_\|,D_\perp,\theta,\phi)_\mathrm{O} = (R_1,R_2,D_\|,D_\perp,\theta,\phi)_\mathrm{P} + \lambda\cdot(0.1\cdot R_{1,\mathrm{P}}, 0.1\cdot R_{2,\mathrm{P}}, 0.1\cdot D_{\|,\mathrm{P}}, 0.1\cdot D_{\perp,\mathrm{P}}, 0.02\pi, 0.02\pi)$, where the subscript P identifies the variable as belonging to the "parent" basis, and $\lambda$ is a normally distributed random number. Such perturbation is introduced in order to assess the stability of the original "parent" solution;
4. Step 2 is repeated for the new "offspring" basis;
5. The "parent" and "offspring" solutions compete, and only the one associated with lower fitting residuals survives. The set formed by the extant nodes and their corresponding $w_\mathrm{n}$ weights defines the new "parent" solution;
6. Steps 3 through 5 are repeated a total of 500 times, and the surviving set is recorded. This directed iterative approach aids the exploration of our vast acquisition space, and facilitates the search of the local solution that better fits the measured data.

The above scheme was implemented for $10^3$ bootstrap samples containing 1500 points randomly sampled with replacement from the measured **s**. Such procedure yields $10^3$ different realizations of $P(R_1,R_2,D_{iso},D_\Delta,\theta,\phi)$, all of which are a valid solution of Eq. (8)[4-6]. The average of the various realizations offers a stable solution to Eq. (S1), wherein the influence of experimental noise and spurious peaks is smoothed out [1,6]. Furthermore, confidence intervals can be estimated from the statistics of the different bootstrap samples [4,5].

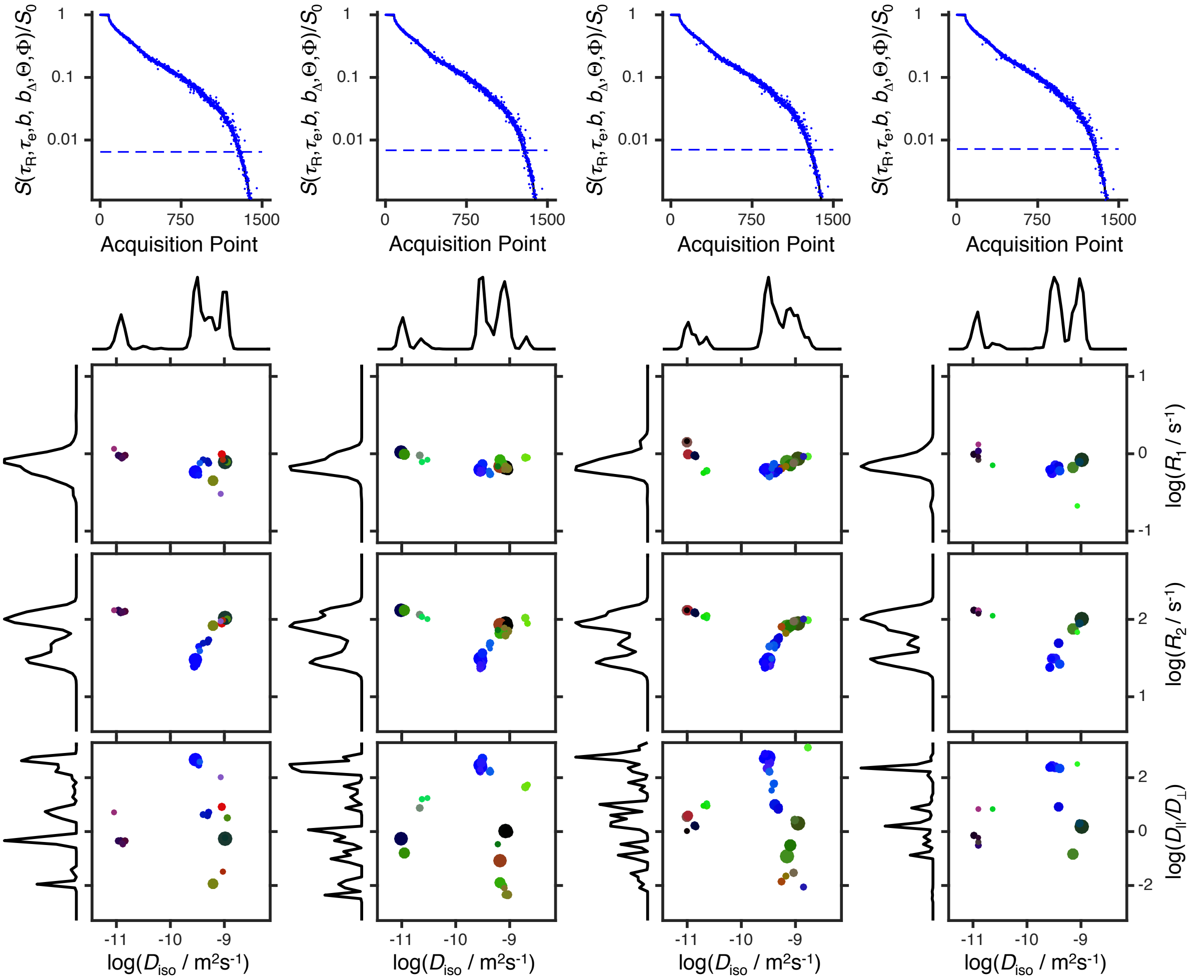


**Figure S1**. Different $P(R_1,R_2,D_{iso},D_\Delta,\theta,\phi)$ distributions estimated from distinct bootstrap samples of the same experimental data set. Each column shows a different solution to Eq. (8) from the main text, all of which were obtained through the unconstrained inversion detailed above. The top panel displays the water signal decay (black line, experimental; blue dots, fitting results; dashed blue line, estimated noise level). Instead of contour plots, $P(R_1,R_2,D_{iso},D_\Delta,\theta,\phi)$ is reported as a set of 2D color-coded scatter plots, where the color-coding defines the orientation distribution of the underlying node (red = $x = \cos\phi\sin\theta$; green = $y = \sin\phi\sin\theta$; blue = $z = \cos\theta$). The size and brightness of each point are proportional to the weight $w_n$ and anisotropy, respectively, of its corresponding node. The lines on the top and at the side of the scatter-plots denote 1D projections of the $(R_1,R_2,D_{iso},D_\Delta)$ space. Notice how distinct solutions return noticeably different distributions without a marked effect on the

fitting quality. This non-unique behavior evidences the ill-conditioned nature of the problem, and shows why model-free solutions should be analyzed carefully.

## UNIFORM VS. NON-UNIFORM SAMPLING SCHEMES

The performance of uniform sampling schemes was studied with data simulated using uniform acquisition protocols with different sampling densities and SNR values. Three distinct sampling grids were tested: a first one with 3×3×4×2 values of ($\tau_R$,$\tau_e$,$b$,$b_\Delta$) and 7 directions ($\Theta$,$\Phi$); the second consisting of a 3×3×5×3 ($\tau_R$,$\tau_e$,$b$,$b_\Delta$) grid spanned over 12 values of ($\Theta$,$\Phi$); the third one formed by 3×4×6×4 ($\tau_R$,$\tau_e$,$b$,$b_\Delta$) values and 18 orientations ($\Theta$,$\Phi$). The $P(D_{iso},D_\Delta)$ projections estimated from the model-free inversion of the simulated data are displayed in Fig. S2. All the displayed ($D_{iso}$,$D_\Delta$) maps have an artefact characterized by a fast isotropic diffusion, whose influence is reduced for higher sampling sizes or SNR values. The persistence of the artefact was not observed for the pseudo-random sampling scheme, as the spurious populations seen in Fig. 6 of the main text vanish at higher sample sizes or SNR levels.

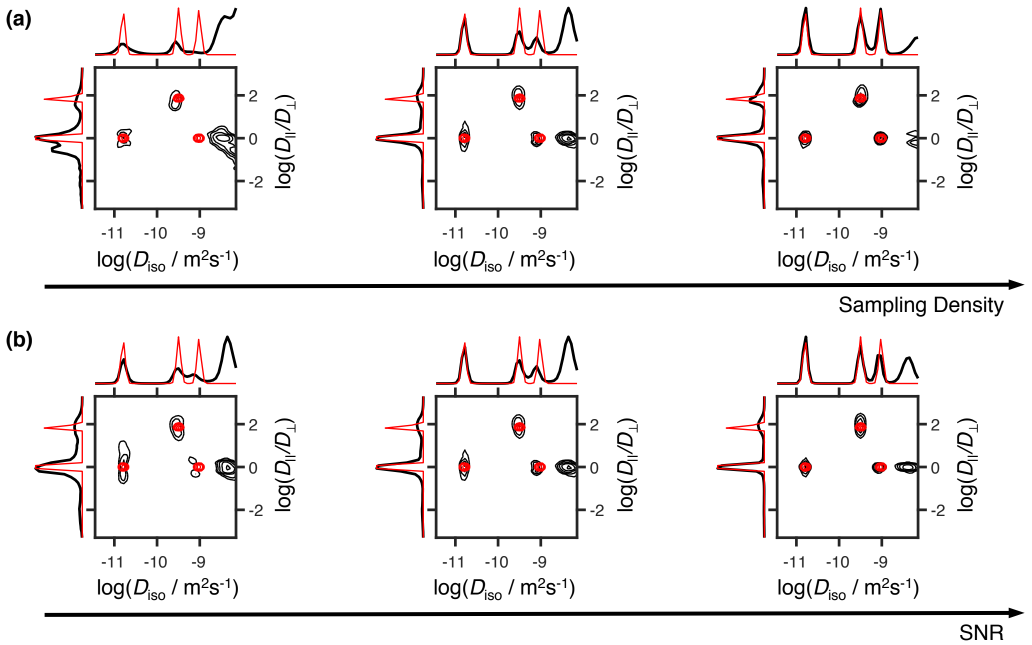


**Figure S2**. Estimation of $P(R_1,R_2,D_{iso},D_{||}/D_\perp,\theta,\phi)$ for artificial data generated on a rectangular sampling grid. The various contour plots show the 2D projections of the ground-truth (red line) and the full inverted distribution (black line) onto the ($D_{iso}$, $D_{||}/D_\perp$) space. (a) Contour plots showing the inversion of data simulated with varying number of acquisition points (from left to right: 504 points, 1620 points, 5184 points) and a constant SNR of 120. (b) Distributions estimated for various levels of SNR (from left to right: SNR = 60, SNR = 120, SNR = 400) and a constant sample size of 1500 points.

The comparison between sampling protocols is further extended in Fig. S3, which shows histograms of statistical measures from the estimated $P(R_1,R_2,D_{iso},D_\Delta,\theta,\phi)$ distribution. There, we can compare the bootstrap distributions for the datasets displayed in Figs. 6a and S2a. Overall, the values estimated from the pseudo-random acquisition protocol are closer to the underlying ground-truth and possess a lower uncertainty. At lower sample sizes (~500 points), the pseudo-random grid has a poor performance, with the estimated solutions having a very high dispersion. However, the variability of the solution space of the non-uniform dataset quickly decreases with increasing sampling densities and, at 1500 data points most of the features of the recovered distribution are within a standard deviation of the ground-truth. Such evolution is not observed for the uniform designs, with ~5000 points still being insufficient to obtain statistical measures within a standard deviation of the underlying "true" values. It should be noted that both sampling schemes yield similar fitting errors.

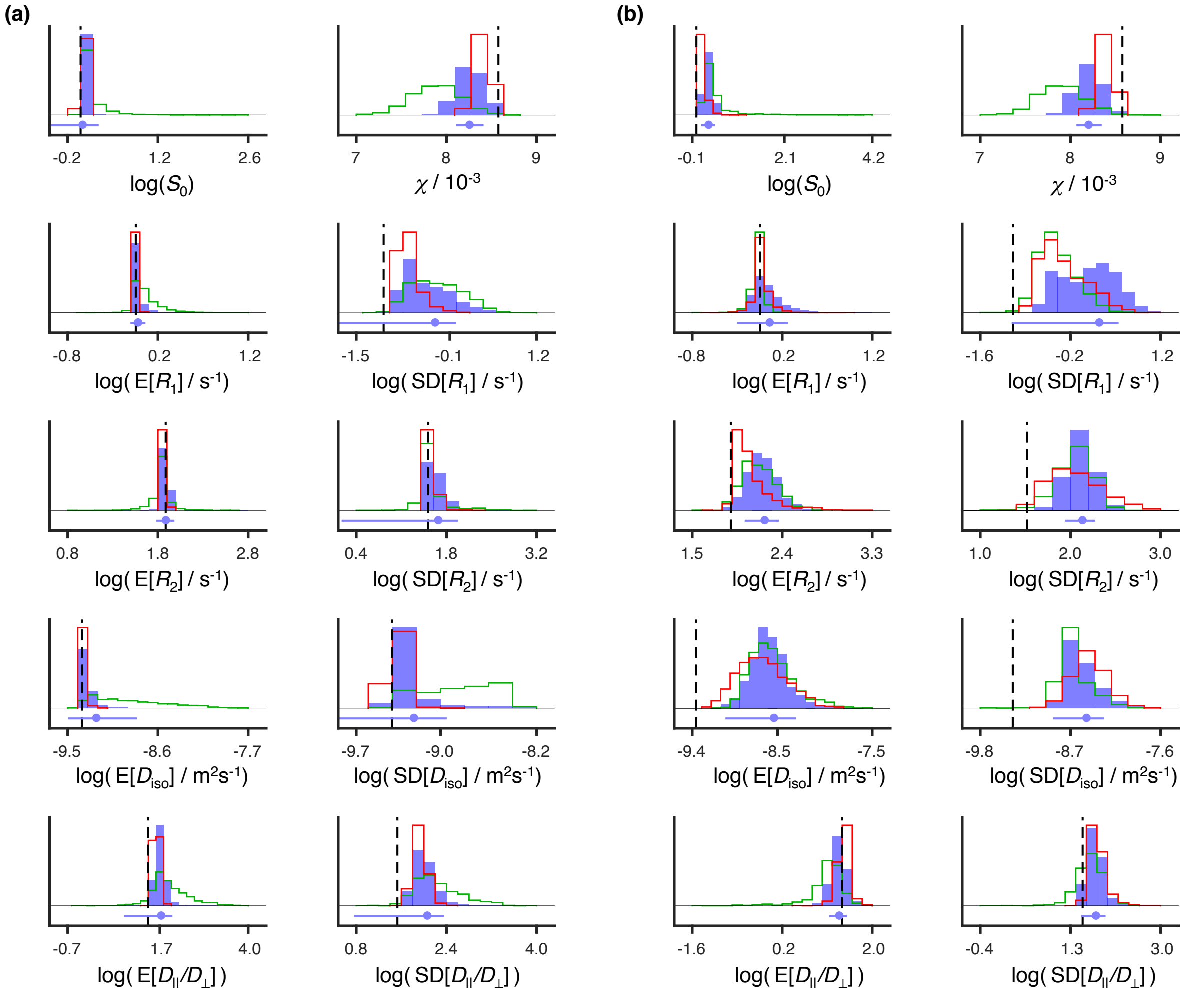


**Figure S3**. Bootstrap statistics of the $P(R_1,R_2,D_{iso},D_{\|}/D_{\perp},\theta,\phi)$ distribution estimated from data simulated using distinct sampling schemes. The histograms were computed from 1000 solutions of Eq. (8) and show the distributions of $R_1$, the root-mean-square error of the fit ($\chi$), and the means (E[$X$]) and standard deviations (SD[$X$]) of $R_1$, $R_2$, $D_{iso}$, and $D_{\|}/D_{\perp}$. Both E[$X$]

and SD[$X$] were calculated for the entire 6D volume of $P(R_1,R_2,D_{\text{iso}},D_{\|}/D_{\perp},\theta,\phi)$. Columns (a), and (b) contain the statistics of data simulated with a pseudo-random scheme and a regular scheme, respectively. The various colours showcase values estimated from artificial datasets with different sampling densities: [green,blue,red] = [500 points, 1500 points, 5000 points] for column (a); [green,blue,red] = [504 points, 1620 points, 5184 points] for column (b). The black dotted line displays the ground truth. The blue dot and line underneath the histogram bars indicate the mean and standard deviation, respectively, of the bootstrap distributions from the datasets with 1500 points and 1620 points. Histograms from the same variable have a constant bin width across the various columns.

## REFERENCES


1 de Almeida Martins, J. P. & Topgaard, D. Two-dimensional correlation of isotropic and directional diffusion using NMR. *Physical review letters* **116**, 087601 (2016).

2 Topgaard, D. Multidimensional diffusion MRI. *Journal of Magnetic Resonance* **275**, 98-113 (2017).

3 Lawson, C. L. & Hanson, R. J. *Solving least squares problems*. (SIAM, 1995).

4 Prange, M. & Song, Y.-Q. Quantifying uncertainty in NMR T2 spectra using Monte Carlo inversion. *Journal of Magnetic Resonance* **196**, 54-60 (2009).

5 Prange, M. & Song, Y.-Q. Understanding NMR T2 spectral uncertainty. *Journal of Magnetic Resonance* **204**, 118-123 (2010).

6 de Kort, D. W., van Duynhoven, J. P., Hoeben, F. J., Janssen, H. M. & Van As, H. NMR nanoparticle diffusometry in hydrogels: enhancing sensitivity and selectivity. *Analytical chemistry* **86**, 9229-9235, doi:10.1021/ac502211q (2014).
